# Sex Differences in Multimorbidity, Inappropriate Medication and Adverse Outcomes of Inpatient Care: MoPIM Cohort Study

**DOI:** 10.3390/ijerph20043639

**Published:** 2023-02-18

**Authors:** Marisa Baré, Marina Lleal, Daniel Sevilla-Sánchez, Sara Ortonobes, Susana Herranz, Olivia Ferrandez, Celia Corral-Vázquez, Núria Molist, Gloria Julia Nazco, Candelaria Martín-González, Miguel Ángel Márquez, on behalf of the MoPIM Study Group

**Affiliations:** 1 Institutional Committee for the Improvement of Clinical Practice Adequacy, Clinical Epidemiology and Cancer Screening Department, CRiSP Research Group, Parc Taulí Hospital Universitari, Institut d’Investigació i Innovació Parc Taulí (I3PT), 08208 Sabadell, Spain; 2 Research Network on Health Services in Chronic Patients (REDISSEC), Instituto de Salud Carlos III, 28029 Madrid, Spain; 3 Research Network on Chronicity, Primary Care and Health Promotion (RICAPPS), ISCIII, 28029 Madrid, Spain; 4 Department of Paediatrics, Obstetrics and Gynaecology, Preventive Medicine and Public Health, Autonomous University of Barcelona, Bellaterra, 08193 Barcelona, Spain; 5 Pharmacy Department, Parc Sanitari Pere Virgili, 08023 Barcelona, Spain; 6 Pharmacy Department, Parc Taulí Hospital Universitari, Institut d’Investigació i Innovació Parc Taulí (I3PT), Universitat Autònoma de Barcelona, 08208 Sabadell, Spain; 7 Acute Care Geriatric Unit, Parc Taulí Hospital Universitari, Institut d’Investigació i Innovació Parc Taulí (I3PT), Universitat Autònoma de Barcelona, 08208 Sabadell, Spain; 8 Pharmacy Department, Consorci Parc de Salut MAR, 08003 Barcelona, Spain; 9 Geriatrics Department-C3RG Research Ggoup, Consorci Hospitalari de Vic, 08500 Vic, Spain; 10 Pharmacy Department, Hospital Universitario de Canarias, 38320 La Laguna, Spain; 11 Internal Medicine Department, Hospital Universitario de Canarias, 38320 La Laguna, Spain; 12 Geriatrics Department, Consorci Parc de Salut MAR, 08003 Barcelona, Spain

**Keywords:** sex perspective, multimorbidity, network analysis, potentially inappropriate medication, adverse drug reaction, in-hospital mortality, outcomes of care

## Abstract

There is no published evidence on the possible differences in multimorbidity, inappropriate prescribing, and adverse outcomes of care, simultaneously, from a sex perspective in older patients. We aimed to identify those possible differences in patients hospitalized because of a chronic disease exacerbation. A multicenter, prospective cohort study of 740 older hospitalized patients (≥65 years) was designed, registering sociodemographic variables, frailty, Barthel index, chronic conditions (CCs), geriatric syndromes (GSs), polypharmacy, potentially inappropriate prescribing (PIP) according to STOPP/START criteria, and adverse drug reactions (ADRs). Outcomes were length of stay (LOS), discharge to nursing home, in-hospital mortality, cause of mortality, and existence of any ADR and its worst consequence. Bivariate analyses between sex and all variables were performed, and a network graph was created for each sex using CC and GS. A total of 740 patients were included (53.2% females, 53.5% ≥85 years old). Women presented higher prevalence of frailty, and more were living in a nursing home or alone, and had a higher percentage of PIP related to anxiolytics or pain management drugs. Moreover, they presented significant pairwise associations between CC, such as asthma, vertigo, thyroid diseases, osteoarticular diseases, and sleep disorders, and with GS, such as chronic pain, constipation, and anxiety/depression. No significant differences in immediate adverse outcomes of care were observed between men and women in the exacerbation episode.

## 1. Introduction

Life expectancy has been progressively increasing in both women and men. Demographic and health changes have contributed to the fact that nowadays more than one in five people in Europe are over 64 years old, and projections point to a continued, gradual growth [1]. Furthermore, the rate of over-ageing (>85 years) also continues to rise, mainly represented by women.

Reaching the age of 65 usually means becoming part of the older adult population. In the ageing process there is an increase in chronic health conditions; however, this process may be so varied that it could be said that there are many different ways to grow old and ill. At the onset of ageing, certain circumstances (such as retirement, offspring moving out, widowhood, or loss of purchasing power) can act as health conditioning factors. In addition, sex is linked to certain differences in terms of health, either independently or through interaction with the previously mentioned factors [2,3]. Sex differences exist regarding certain indicators of morbidity, with different disease incidences and higher odds of prevalent comorbidity in women (OR 1.11, 95% CI 1.07–1.15), as well as in mortality in general and avoidable mortality. Mortality rates from treatable causes were about 40% higher in men and preventable mortality rates were 2.6 times higher in men from OECD countries in 2019 [4,5,6].

On another note, multiple chronic health problems in the same individual (known as multimorbidity) can constitute profiles or patterns, some of which have already been described in older patients [7,8]. Multimorbidity, together with geriatric syndromes and frailty, frequently coexisting in the ageing process, increase clinical complexity and can lead to significant medicalization [9]. In these circumstances, there is an increased likelihood of low-value clinical practices, such as excessive polypharmacy and potentially inappropriate prescribing (PIP) [10]. These practices, in turn, may be determinant of certain adverse drug reactions (ADRs) or even mortality. In this context, differences have been described between older men and women in multimorbidity and PIP [11,12,13,14], although it is unknown whether these differences are accompanied by differences in immediate adverse outcomes of inpatient care.

Provision of health care services based on sex is generally focused on health problems or conditions linked to the reproductive system. Therapeutic guidelines for most chronic health problems do not include a comprehensive approach depending on sex (biological) or gender (sociocultural); this is even less so in older patients, due in part to their under-representation in clinical trials [15]. Furthermore, the consideration of interventions aimed at situations of multimorbidity instead of isolated diseases is quite recent [16], partly because of limited evidence on its effectiveness [17,18,19].

To date, there is no published evidence on the possible differences in multimorbidity, geriatric syndromes, PIP, and adverse outcomes of care, simultaneously, from a sex perspective in older patients.

Taking into account all the previous considerations, in the context of the MoPIM study, which evaluated different clinical aspects of older inpatients, we developed the present analyses. The objectives were to identify possible differences in multimorbidity and PIP between older women and men admitted to hospital because of a chronic disease exacerbation, as well as to compare certain immediate in-hospital adverse outcomes.

## 2. Materials and Methods

### 2.1. Design and Setting

A multicenter, prospective cohort study including older patients hospitalized at the internal medicine or geriatric services at five general teaching hospitals in three different regions of Spain between September 2016 and December 2018 was designed. The detailed protocol was previously published [20].

For the purposes of the study, older patients (≥65 years old) admitted as a result of the exacerbation of their chronic pathology, according to the attending physician’s judgement, were included. Patients referred to home hospitalization, admitted because of an acute process not related to any chronic disease, or with a fatal outcome expected at the time of admission were not included. No written informed consent was deemed necessary for this study.

### 2.2. Data Acquisition and Variables

The following sociodemographic and clinical data were retrieved from the electronic health records by the clinical team responsible for the patient: patient’s code, date of birth, sex, date of admission to hospital, date of discharge, discharge to a nursing home, functional status just before entering the hospital (Barthel index) [21], household (alone, with relatives or other people, in a nursing home), tobacco consumption (non-smoker, former smoker, smoker), alcohol consumption (non-drinker, former drinker, heavy drinker), and the existence of a prior exacerbation that motivated utilization of healthcare services (including primary care, emergencies, hospital admission, outpatient care, or home care) in the 3 months prior to hospitalization. Frailty was assessed as usual in the centers: two departments used the recently developed scale Frail-VIG [22], while the others considered clinical judgement (although based on the same variables). Active chronic conditions (CCs) of the patient at hospital arrival, including some risk factors (hypertension, obesity, hip fracture or other fractures, osteoporosis, dyslipidemia), as well as specific geriatric syndromes (GSs), were collected according to a consensual list defined in the study protocol [20]. A condition was considered chronic when it lasted for at least 6 months, including past conditions that require ongoing disease or risk management, important conditions with a significant risk of recurrence, or past conditions that have continuing implications for patient management, as defined by Salisbury and colleagues [23].

Regarding pharmacological variables, the number of chronic medications in the electronic prescribing system at admission, all potentially inappropriate medications (PIMs) detected upon admission according to STOPP criteria 2nd version (Screening Tool of Older Person’s potentially inappropriate Prescriptions), and all potentially omitted prescriptions (PPOs) according to START criteria 2nd version (Screening Tool to Alert doctors to Right Treatment) were collected by the pharmacist of the team, as part of the usual patient care routine [24,25]. The STOPP/START criteria were the first European criteria and are currently the most used and validated in European older people. According to the study criteria, a medication was considered chronic if prescribed at least 3 months before admission, while creams, ointments, healing material, and over-the-counter medicines were not considered. All STOPP/START criteria were assessed, except for START criteria I (vaccines), due to difficulties of some centers in accessing the information.

All adverse drug reactions (ADRs) detected at admission or occurring during hospitalization until discharge or death were collected by the clinical team. An ADR was defined as a response to a drug which is noxious and unintended, and which occurs at doses normally used in man for prophylaxis, diagnosis, or therapy of disease or for the modification of physiologic function, according to the World Health Organization and the European Medicines Agency [26,27]. Consequences in terms of health (death, life-threatening, lengthening of hospitalization, or other clinically important consequences under medical criteria) were registered if the ADR appeared during the hospital stay.

The analyzed outcomes were: length of stay (LOS); discharge to nursing home; in-hospital mortality; cause of in-hospital mortality (disease exacerbation, treatment complication, or other causes (unforeseeable and unrelated to the disease or the treatment)); existence of any ADR detected at admission or during hospitalization; and worst consequence of the ADRs detected during hospitalization.

### 2.3. Sampling and Analysis

A consecutive sample of 740 patients was included, according to the study protocol criteria [20].

Some CCs were grouped according to the clinical criteria detailed in a previous publication [7]. Finally, 50 CCs and 14 GSs were analyzed (CCs are shown in Tables S1–S3, GSs in Table S4). The updated Charlson Comorbidity Index [28], age adjusted, was computed and categorized according to the tertile distribution.

Age was grouped into decades (65–74 years, 75–84 years, 85–94 years, ≥95 years) and into two groups (65–84 years, and ≥85 years) and the Barthel index was grouped as in Mahoney et al. (<20 total dependence, 20–35 severe dependence, 40–55 moderate dependence, 60–95 low dependence, 100 independent) [29]. For the purposes of these analyses, the number of chronic medications was also categorized as follows: oligopharmacy (0–4), moderate polypharmacy (5–9), and excessive polypharmacy (≥10). There were no missing data, except from tobacco and alcohol consumption, for which a “Not available” category was created.

Descriptive statistics were derived to obtain overall prevalence estimates of all variables. In order to describe relationships between pairs of both CC and GS, a network graph was created for each sex, using ForceAtlas2 graph layout algorithm [30] from Gephi open source software version 0.9.2 [31]. Data was filtered by >2% prevalence and by >2 pairwise observed/expected (O/E) ratio.

Bivariate analyses were conducted to assess possible associations between sex (woman and men) and sociodemographic variables, CC (including risk factors), GS, all variables related to inappropriate medication, and all outcomes, by the chi-square test for categorical variables or the Wilcoxon test for continuous variables. A stratified analysis by age group (65–84 years, ≥85 years) was also performed for all the variables. Odds ratios and their confidence intervals (95% CI) were calculated for those statistically significant relationships and were displayed together in a forest plot by sex. All descriptive and bivariate analyses were performed with R [32].

## 3. Results

A consecutive sample of 740 patients was included, with a mean age of 84.1 years (median 85, interquartile range 80–89), and 53.2% (n = 394) were females. Among all patients, 53.5% were 85 years old or over.

The distribution of the main clinical variables and adverse outcomes stratified by sex and by the two age groups is shown in Table 1. A higher percentage of advanced age women was found, as well as a higher proportion of women living alone (18.53% vs. 14.16%) or in nursing homes (15.48% vs. 9.83%), especially in those over 84 years old. Women presented a higher level of dependence according to the Barthel index and a higher percentage of frailty (OR 1.81, 95% CI 1.34–2.44). Both current or past smoking and alcoholism were more frequently identified in men.

**Figure 1:**
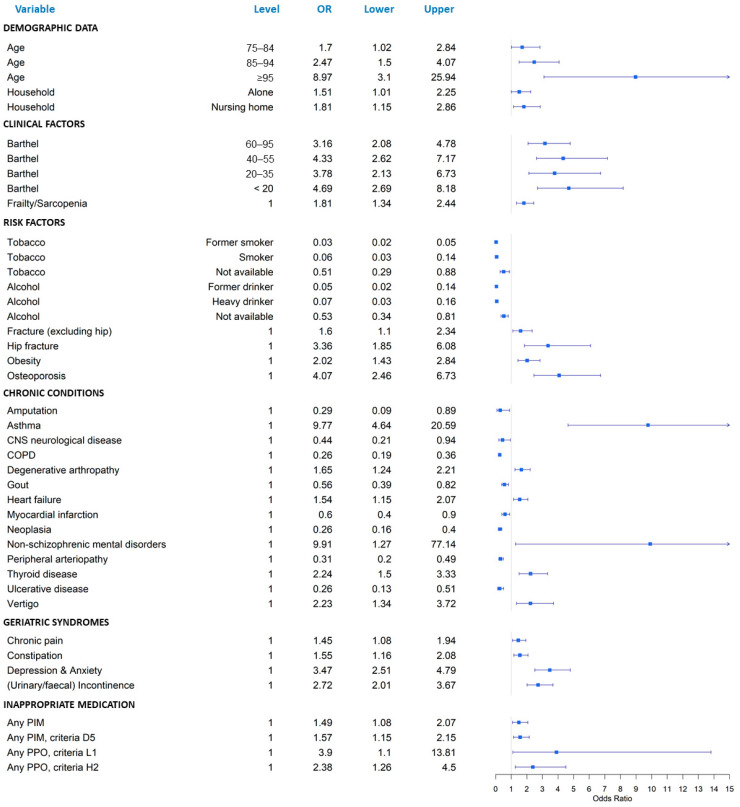
Forest plot of significant clinical and sociodemographic variables. Odds ratios and their upper and lower 95% confidence intervals are shown. Reference category: men. CNS: central nervous system. COPD: chronic obstructive pulmonary disease. PIM: potentially inappropriate medication. PPO: potential prescribing omission.

No significant differences were observed between men and women in the Charlson index. Nonetheless, as shown in Figure 1 and Tables S1–S4, the distribution of some CCs and GSs was indeed different. Thus, women were more likely to present certain risk factors, such as a history of hip fracture (OR 3.36; 95% CI 1.85–6.08), obesity (OR 2.02; 95% CI 1.43–2.84) or osteoporosis (OR 4.07; 95% CI 2.46–6.73), compared to men. In addition, women were more likely to suffer from certain non-schizophrenic mental diseases (OR 9.91; 95% CI 1.27–77.14), asthma (OR 9.77; 95% CI 4.64–20.59), thyroid diseases (OR 2.24; 95% CI 1.5–3.33), vertigo (OR 2.23; 95% CI 1.34–3.72), degenerative arthropathy (OR 1.65; 95% CI 1.24–2.21), or heart failure (OR 1.54; 95% CI 1.15–2.07). Other diseases such as chronic obstructive pulmonary disease (COPD), and neoplasia or peripheral arteriopathy, were significantly less frequent. Among the different GSs, depression/anxiety (OR 3.47; 95% CI 2.51–4.79), incontinence (OR 2.72; 95% CI 2.01–3.67), constipation (OR 1.55; 95% CI 1.16–2.08), and chronic pain (OR 1.45; 95% CI 1.08–1.94), were significantly more frequent in women.

**Figure 2:**
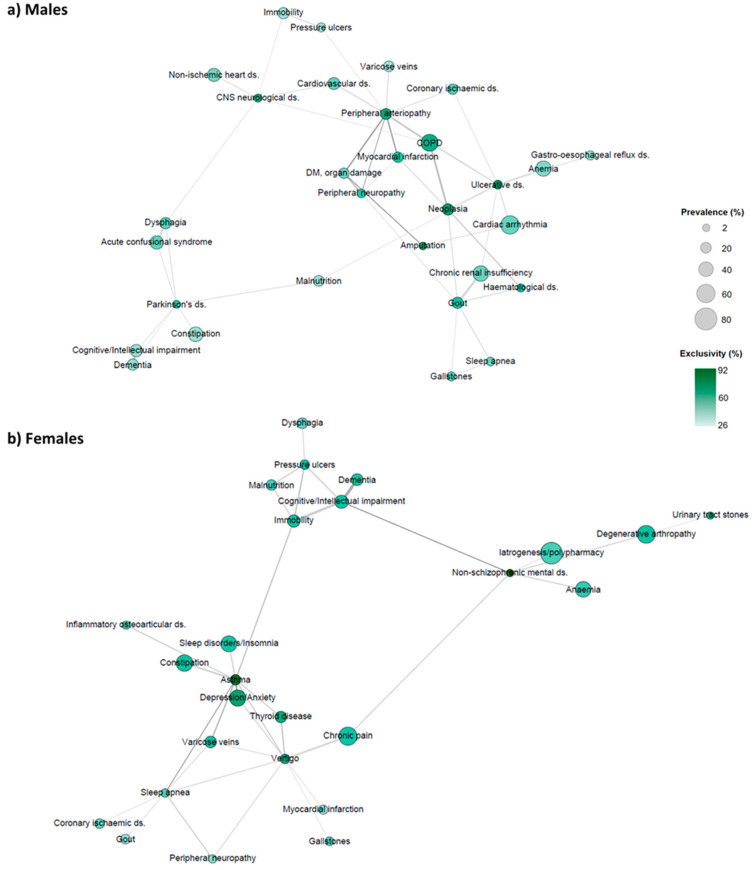
Network visualization of chronic conditions and geriatric syndromes using the ForceAtlas2 graph layout algorithm. Data was filtered by >2% prevalence and by >2 pairwise O/E ratio. The main components of networks are nodes (chronic diseases/geriatric syndromes) and edges (coexistence of diseases or syndromes) that connect nodes in the network. Edge width is proportional to the prevalence of each chronic disease pair. Edge color is proportional to the Observed/Expected (O/E) ratio of the pair of diseases (a darker edge means a higher O/E ratio). The size of the nodes is proportional to the prevalence of the disease. The intensity of the color is proportional to the sex exclusivity. CNS: central nervous system; COPD: chronic obstructive pulmonary disease; DM: diabetes mellitus; ds: disease. Figure 2a includes male patients; Figure 2b includes female patients.

Likewise, Figure 2 shows the prevalence and exclusivity of the strongly related pairs of CC and GS (>2 pairwise O/E ratio) for each sex separately. According to the figure, the most inter-connected CCs or GSs differed between sexes. In women, for example, significant associations between CCs, such as asthma, vertigo, thyroid diseases, osteoarticular diseases, and sleep disorders, as well as with GSs, such as chronic pain, constipation, and anxiety/depression, were present. Regarding men, connections involving peripheral arteriopathy and other cardiovascular and neurological diseases were found, along with COPD and neoplasms or even gout.

Both men and women, regardless of age group, had a similar frequency (around 70%) of exacerbation and consultation with health services in the three previous months.

Concerning chronic medication, around 95% of the patients had polypharmacy, with no notable differences between men and women. As shown in Table 1 and Figure 1, the existence of at least one PIM was significantly higher in women (76.9% vs. 69.1%; OR 1.49; 95% CI 1.08–2.07), but not the existence of at least one PPO. Specifically, the STOPP criteria D5 (benzodiazepines for 4 or more weeks) and L1 (use of transdermal strong opioids as first line therapy for mild pain), as well as the START criterion H2 (laxatives in patients receiving opioids regularly) were the only ones that were significantly more frequent in women (see Figure 1 and Tables S5 and S6).

Around a third of the patients (245/740) presented at least an ADR. A share of 16.2% (120/740) of patients had an ADR during hospitalization and a 20.7% (153/740) had an ADR detected at the time of admission. About 14% of ADRs detected during hospitalization entailed some threat to life in women, whereas this was about 5% in men. No deaths were attributable to ADRs. Ultimately, 8.9% (n = 66) of the patients died at the hospital.

Regarding the comparison of adverse outcomes, none showed statistically significant differences between men and women. Nevertheless, the stratified analyses showed differences in the oldest women (>84 years), which were more frequently referred to nursing homes (21.6% vs. 12.8%), compared to the oldest men.

## 4. Discussion

### 4.1. Main Important Results and Novelty

The results of this study indicate that older women have a clearly different multimorbidity profile (when considering chronic conditions and geriatric syndromes) from older men. Women seemed to have more frailty and more dependence in all age strata. Furthermore, women had near a 50% higher probability of having a potentially inappropriate prescription, more specifically due to STOPP criteria related to benzodiazepines (D5) and opioids (L1), as well as to the START criterion H2, which considers the lack of laxatives when taking opioids regularly. Around one-third of the patients presented at least an ADR and 16% had a new ADR during the hospitalization episode, but no significant differences were observed in the existence of an ADR nor the worst consequence of ADRs between sexes. No significant differences were observed in the in-hospital mortality (8.9%) of the exacerbation episode between women and men.

To our knowledge, this is the first study that graphically captures the interrelationship between different CCs and GSs, evaluates PIMs and PPOs using explicit criteria and, simultaneously, quantifies ADRs and their consequences in a cohort of older men and women treated in different hospitals. It provides an overview, but addresses several clinical aspects of interest in older patients, comparing women and men.

### 4.2. Comparing with Other Studies

The number of publications describing different aspects of multimorbidity has grown considerably in the last decade. Unfortunately, comparison between them is difficult due to the significant variability in methodology and age groups considered [33,34]. Nonetheless, some authors have published interesting results on the differences in morbidity, polypharmacy, and/or treatment inadequacy between older men and women. For example, Maxwell et al., 2021 described greater multimorbidity and polypharmacy in elderly women in the community [11]. In Ahrenfeldt’s 2019 study, based on data from the Survey of Health, Aging and Retirement in Europe, authors identified greater comorbidity and frailty in women, with differences becoming more evident at older ages [5]. Almagro et al., 2020, in a cohort of hospitalized older patients, also described different patterns of multimorbidity between sexes, although no differences were observed in outcomes such as mortality or readmissions per year [12]. Despite the difficulties in comparing profiles or patterns between different studies, in Almagro’s article, with a similar cohort, authors identified that conditions such as dementia or heart failure, and a neurological or osteoarticular pattern, might be more frequent in hospitalized women, while cancer or a respiratory pattern predominated in men. Robertson et al., 2022, in a study of hospitalized adults, found a higher prevalence in women in a pattern labelled as ‘chronic pain’ [35].

Furthermore, Toepfer et al. in 2019 and more recently in 2022 described a higher prevalence of polypharmacy and PIP in women, despite not being clear whether sex was the determining factor or whether other conditions, such as frailty or educational level, differed between men and women [36,37]. According to the recent systematic review of Delara et al., sex was not clearly associated with polypharmacy [38]. In an article by Muhlack, sex was identified as a determining factor in the existence of PIP, apart from age, frailty, or some geriatric syndromes [39]. A work by Johnell et al. in 2009 suggested that the greater percentage of PIPs analyzed in women was not due to socio-economic or morbidity differences [40]. Nevertheless, in a recently published paper of the MoPIM project, we were not able to identify sex as an independent factor associated with the most frequent inappropriate medication [25].

### 4.3. Clinical Interpretation of Results

According to our results, there are important clinical differences between older men and women. The identified profile of older women, i.e., more fragile and dependent, could be explained by their morbidity [41]. In fact, the risk of frailty in older women may be between 34% and 144% higher than that in older men. This observed difference at a given moment can be the consequence of multiple past or present factors, and their analysis differs from the objectives of this study. However, some present situations, such as depressive or anxious states or a history of fracture, which are more prevalent among women in this cohort, could be a triggering factor for their greater frailty. Moreover, the results of our study show that older women could have a 5% to 100% higher risk of being prescribed any PIM, which could also have some consequences on their frailty and dependence.

The different multimorbidity networks observed in men and women summarize the relationships between chronic conditions and, at the same time, show connections that could have the same physiopathological basis or even a causal relationship. For example, in women, osteoarticular conditions may cause chronic pain which, depending on the individual resilience, could cause some adaptation difficulties. This situation could lead to depressive states, anxiety, insomnia, or vertigo. In men, COPD, diabetes mellitus and its related vascular pathology could be attributable, to a greater or lesser extent, to the greater prevalence of risk factors (such as tobacco and alcohol, among others). It is worth mentioning that adding geriatric syndromes in our analyses provides a more complete and comprehensive view of the possible patient preventive (especially in not-yet-senile individuals) and therapeutic needs.

By comparison, some of the clinical conditions interrelated in women according to the network analysis, such as chronic pain, insomnia, degenerative arthropathy, anxiety, and depressive states, may simultaneously favour risk factors such as obesity and osteoporosis, or could lead to medicalized situations that may require another type of therapeutic approach. Furthermore, over-medicalization could even be a determining factor in other health problems. In fact, the inappropriate use of benzodiazepines or opioids can increase the risk of fractures [42]. In addition to the clinical differences found, we observed that more women were living in nursing homes. The lack of connection in the information system between health centres and nursing homes could hinder a timely review of inappropriate medication and an adequate medication reconciliation.

However, despite these significant differences between older men and older women, the data show that women did not have clearly worse adverse outcomes than men in the evaluated hospitalization episode, at least in terms of LOS, in-hospital mortality, or ADRs. Although a relationship between PIP and ADRs has been described in the literature and women seem to have a higher probability of ADRs [43], the higher probability of chronic PIP in women was not accompanied by a higher probability of ADRs in our study. This lack of relationship was independent of whether the ADRs were detected at the time of admission (which would be related to the patient’s basal medication before arriving at the hospital) or if they were detected throughout the process of hospitalization (where other clinical factors due to the acute situation may have had an impact). In fact, the medication review process carried out during the hospitalization period should have led to minimizing the appearance of ADRs during hospital stay. Nevertheless, it is possible that the inclusion criteria of the study did not allow the detection of possible differences that could be detected in a community setting, or that it is necessary to go deeper into the active principles involved as well as the possible persistence of PIP during the hospital stay, an approach that was not part of the objectives of these analyses.

It has been also described that women might be more susceptible to drug-related harm and ADRs because of physiological differences [44]. Although no statistically significant differences in ADR indicators were detected in our research, women suffered more life-threatening situations attributable to ADRs than men. This fact must surely have had an impact on the process of care and on other clinical outcomes not considered in this research.

### 4.4. Possible Clinical Implications

Although the evaluated outcomes did not show significant differences in the stratified analysis, and that it would be necessary to have much more data on the hospital care process in order to explain these findings, other noteworthy results of these analyses highlight substantial differences between men and women that might suggest possible actions or proposals.

Incorporating a more comprehensive view of patient chronic morbidity, functional status, and its therapeutic implications, taking into account the common types of connections among different clinical conditions, should facilitate the development of clinical practice guidelines that consider a gender or sex perspective in older patients and also incorporate patient-defined outcomes, which could be different between men and women.

As has been described, there is no clear evidence to date on the effectiveness of clinical interventions or organizational models aimed at patients with multiple conditions [19], and it is necessary to go deeper into the aspects that differentiate men and women regarding medication and its adverse reactions [45]. For this reason, further studies similar to ours will be needed, which at the same time incorporate more relevant clinical aspects, in order to corroborate some of our observations. Additionally, it would be desirable to continue exploring possible care models focused on prevention (early detection of risk situations) that take into account the possible differences between subgroups of patients, in this case men and women. Furthermore, it may even be appropriate to strengthen the sex or gender perspective in social and economic policies, in order to ensure that women enjoy better autonomy and less frailty and medicalization during their aging.

### 4.5. Strengths and Limitations

Together with these interesting results, it should be taken into account that patients included in this research would not be representative of older people in the community, but of older patients admitted due to an exacerbation of their chronic conditions. In fact, they might represent a more complex population than the community-dwelling one. Therefore, conclusions can only apply to similar populations. As pointed out in the methodology, frailty was not collected in a standardized way everywhere, but by applying the criteria used by physicians in each center. This could underestimate or overestimate its value but, on the other hand, the measure of frailty in this observational study reflects the real clinical practice in the studied period in different centres. Finally, it is clear that the frequency of ADRs could have been underestimated because their detection, registration, and classification depend on multidisciplinary and proactive work. Nonetheless, the involvement of both clinicians and pharmacists in the study ensured the greatest possible comprehensiveness.

Despite the aforementioned possible limitations, this was a multicenter and multidisciplinary cohort study that focused on both clinical (CCs and GSs) and appropriateness issues, as well as on immediate adverse outcomes and, for that reason, covers a whole range of clinically important issues. In addition, the list of CCs and GSs taken into account was exhaustive, and not limited to the most prevalent ones. Moreover, beyond the simple comparison of single diseases or the counting of important diseases, our analyses show that there are certainly different hubs of clinical conditions, i.e., highly interconnected pathologies (with many significant associations with different conditions), that also differ between women and men. In this sense, these hubs not only highlight the higher or lower prevalence or exclusivity of some pathologies in women and men, but also the interrelationship between pairs of conditions. Thus, this can bring to light other conditions that are also important due to their significant connection. This method has been recently described in a review of analytical methods for identifying patterns of multimorbidity [46].

In this research, explicit criteria (STOPP-START) were applied to assess PIP at the time of admission, as usual, and, at the same time, we were able to detect an ADR in 1 of every 3 patients. A recent review of Jennings et al. regarding in-hospital ADRs in older patients, and a recent work of Robinson EG et al. in community-dwelling older patients, describe percentages of ADRs of 16% and 20%, respectively [47,48].

## 5. Conclusions

In conclusion, older inpatient women present a different profile of morbidity and geriatric syndromes that could be related to their greater frailty and lack of autonomy. Despite no evident differences in polypharmacy of chronic medication, older women have a higher probability of being prescribed potentially inappropriate medication, specifically anxiolytics and pain management drugs. However, no clear differences in the adverse outcomes of inpatient care were found.

## Figures and Tables

**Table 1:** Clinical variables, medication, and adverse outcomes in older patients hospitalized for acute exacerbation of chronic disease. Categorical variables: N (vertical %), chi-squared test. Numerical variable: Median (IQ range), Wilcoxon test. * Percentage and *p*-value calculated over the alive cohort. ** Percentage and *p*-value calculated over the deceased cohort. PIM: Potentially inappropriate medication. PPO: Potential prescribing omission.

Variable	Level	Total Cohort			65–84 Years			≥85 Years		
		Female	Male	*p*-Value	Female	Male	*p*-Value	Female	Male	*p*-Value
Total		394	346		157	187		237	159	
SOCIODEMOGRAPHIC										
Age	<75	29 (7.4)	52 (15.0)	<0.001	29 (18.5)	52 (27.8)	-	-	-	-
	75–84	128 (32.5)	135 (39.0)		128 (81.5)	135 (72.2)		-	-	
	85–94	212 (53.8)	154 (44.5)		-	-		212 (89.5)	154 (96.9)	
	≥95	25 (6.4)	5 (1.5)		-	-		25 (10.6)	5 (3.1)	
Household	Alone	73 (18.5)	49 (14.2)	0.009	35 (22.3)	29 (15.5)	0.272	38 (16.0)	20 (12.6)	0.019
	Nursing home	61 (15.5)	34 (9.8)		12 (7.6)	16 (8.6)		49 (20.7)	18 (11.3)	
	With relatives/other people	260 (66.0)	263 (76.0)		110 (70.1)	142 (75.9)		150 (63.3)	121 (76.1)	
CLINICAL AND RISK FACTORS										
Barthel	<20	59 (15.0)	31 (9.0)	<0.001	18 (11.5)	16 (8.6)	0.001	41 (17.3)	15 (9.4)	<0.001
	20–35	46 (11.7)	30 (8.7)		10 (6.4)	14 (7.5)		36 (15.2)	16 (10.1)	
	40–55	79 (20.1)	45 (13.0)		30 (19.1)	22 (11.8)		49 (20.7)	23 (14.5)	
	60–95	165 (41.9)	129 (37.3)		66 (42.0)	56 (30.0)		99 (41.8)	73 (45.9)	
	100	45 (11.4)	111 (32.1)		33 (21.0)	79 (42.3)		12 (5.1)	32 (20.1)	
Frailty	No	125 (31.7)	158 (45.7)	<0.001	59 (37.6)	98 (52.4)	0.008	66 (27.9)	60 (37.7)	0.05
	Yes	269 (68.3)	188 (54.3)		98 (62.4)	89 (47.6)		171 (72.2)	99 (62.3)	
Charlson	2–5	86 (21.8)	62 (17.9)	0.271	42 (26.8)	44 (23.5)	0.781	44 (18.6)	18 (11.3)	0.089
	6–8	219 (55.6)	192 (55.5)		79 (50.3)	97 (51.9)		140 (59.1)	95 (59.8)	
	9–14	89 (22.6)	92 (26.6)		36 (22.9)	46 (24.6)		53 (22.4)	46 (28.9)	
Tobacco	Non-smoker	325 (82.5)	92 (26.6)	<0.001	133 (84.7)	37 (19.8)	<0.001	192 (81.0)	55 (34.6)	<0.001
	Former smoker	19 (4.8)	197 (56.9)		9 (5.7)	114 (61.0)		10 (4.2)	83 (52.2)	
	Smoker	7 (1.8)	33 (9.5)		5 (3.2)	25 (13.4)		2 (0.8)	8 (5.0)	
	Not available	43 (10.9)	24 (6.9)		10 (6.4)	11 (5.9)		33 (13.9)	13 (8.2)	
Alcohol	Non-drinker	338 (85.8)	197 (56.9)	<0.001	137 (87.3)	87 (46.5)	<0.001	201 (84.8)	110 (69.2)	<0.001
	Former drinker	4 (1.0)	47 (13.6)		2 (1.3)	35 (18.7)		2 (0.8)	12 (7.6)	
	Heavy drinker	6 (1.5)	51 (14.7)		5 (3.2)	41 (21.9)		1 (0.4)	10 (6.3)	
	Not available	46 (11.7)	51 (14.7)		13 (8.3)	24 (12.8)		33 (13.9)	27 (17.0)	
Prior exacerbation	No	126 (32.0)	99 (28.6)	0.361	40 (25.5)	50 (26.7)	0.887	86 (36.3)	49 (30.8)	0.309
	Yes (total)	268 (68.0)	247 (71.4)		117 (74.5)	137 (73.3)		151 (63.7)	110 (69.2)	
INAPPROPRIATE MEDICATION										
Polypharmacy	Oligopharmacy (0–4)	28 (7.1)	18 (5.2)	0.393	5 (3.2)	9 (4.8)	0.27	23 (9.7)	9 (5.7)	0.265
	Moderate polypharmacy (5–9)	142 (36.0)	117 (33.8)		52 (33.1)	48 (25.7)		90 (38.0)	69 (43.4)	
	Excessive polypharmacy (≥10)	224 (56.9)	211 (61.0)		100 (63.7)	130 (69.5)		124 (52.3)	81 (50.9)	
Any PIM	No	91 (23.1)	107 (30.9)	0.021	38 (24.2)	58 (31.0)	0.2	53 (22.4)	49 (30.8)	0.077
	Yes	303 (76.9)	239 (69.1)		119 (75.8)	129 (69.0)		184 (77.6)	110 (69.2)	
Any PPO (no vaccines)	No	252 (64.0)	225 (65.0)	0.821	104 (66.2)	131 (70.1)	0.522	148 (62.5)	94 (59.1)	0.575
	Yes	142 (36.0)	121 (35.0)		53 (33.8)	56 (30.0)		89 (37.6)	65 (40.9)	
ADVERSE OUTCOMES										
Length of stay	-	11 (7–17)	11.5 (8–17)	0.146	13 (8–20)	13 (9–20.5)	0.763	9 (7–15)	10 (7–14.5)	0.642
Nursing home as destination at discharge *	No	294 (82.6)	275 (86.5)	0.199	123 (89.1)	146 (85.9)	0.496	171 (78.4)	129 (87.2)	0.046
	Yes	62 (17.4)	43 (13.5)		15 (10.9)	24 (14.1)		47 (21.6)	19 (12.8)	
In-hospital mortality	No	356 (90.4)	318 (91.9)	0.542	138 (87.9)	170 (90.9)	0.464	218 (92.0)	148 (93.1)	0.832
	Yes	38 (9.6)	28 (8.1)		19 (12.1)	17 (9.1)		19 (8.0)	11 (6.9)	
Cause of in-hospital mortality **	Treatment complication	0 (0)	1 (3.9)	0.388	0 (0)	1 (6.7)	0.501	0 (0)	0 (0)	1.000
	Diseaseexacerbation	32 (86.5)	23 (88.5)		16 (88.9)	13 (86.7)		16 (84.2)	10 (90.9)	
	Other cause	5 (13.5)	2 (7.7)		2 (11.1)	1 (6.7)		3 (15.8)	1 (9.1)	
Any adverse drug reaction (ADR)	No	262 (66.5)	233 (67.3)	0.869	98 (62.4)	122 (65.2)	0.667	164 (69.2)	111 (69.8)	0.985
	Yes	132 (33.5)	113 (32.7)		59 (37.6)	65 (34.8)		73 (30.8)	48 (30.2)	
ADR at admission (n = 153)	No	47 (35.6)	45 (39.8)	0.511	20 (33.9)	28 (43.1)	0.357	27 (37.0)	17 (35.4)	1.000
	Yes	85 (64.4)	68 (60.2)		39 (66.1)	37 (56.9)		46 (63.0)	31 (64.6)	
New ADR during hospitalization (n = 120)	No	69 (52.3)	56 (49.6)	0.702	32 (54.2)	30 (46.2)	0.472	37 (50.7)	26 (54.2)	0.715
	Yes	63 (47.7)	57 (50.4)		27 (45.7)	35 (53.9)		36 (49.3)	22 (45.8)	
Worst consequence of ADR during hospitalization	Life-threatening	9 (14.3)	3 (5.3)	0.240	5 (18.5)	2 (5.7)	0.214	4 (11.1)	1 (4.5)	0.340
	Lengthening of stay	26 (41.3)	28 (49.1)		13 (48.1)	16 (45.7)		13 (36.1)	12 (54.6)	
	Other clinically imp.	28 (44.4)	26 (45.6)		9 (33.3)	17 (48.6)		19 (52.8)	9 (40.9)	

## Data Availability

The data presented in this study are openly available in Zenodo at DOI 10.5281/zenodo.7371151.

## Supplementary Materials

The following supporting information can be downloaded at: https://www.mdpi.com/article/10.3390/ijerph20043639/s1. Table S1: Prevalence of chronic conditions by sex for the total cohort; Table S2: Prevalence of chronic conditions stratified by age group 65–84 years; Table S3: Prevalence of chronic conditions by sex stratified by age group ≥85 years; Table S4: Prevalence of geriatric syndromes by sex for the total cohort; Table S5: Potentially inappropriate medication according to STOPP criteria by sex; Table S6: Potential prescribing omissions according to START criteria by sex.
