# Supplementary material for: Sex Differences in Multimorbidity, Inappropriate Medication and Adverse Outcomes of Inpatient Care: MoPIM Cohort Study
Source: Int J Environ Res Public Health. 2023 Feb 18;20(4):3639. doi: 10.3390/ijerph20043639 (PMC9964600; doi:10.3390/ijerph20043639)
Supplement: Supplementary file 1 [file ijerph-20-03639-s001.zip › ijerph-2044822-supplementary.pdf]

Table S1. Prevalence of chronic conditions by sex for the total cohort. Fisher's exact test. CNS: central nervous system. COPD: chronic obstructive pulmonary disease.

Variable	Level	Female		Male		Total	P-value
		N	%	N	%	N	
Amputation	No	390	98.98	334	96.53	724	0.039
	Yes	4	1.02	12	3.47	16	
Anemia	No	212	53.81	194	56.07	406	0.554
	Yes	182	46.19	152	43.93	334	
Asthma	No	320	81.22	338	97.69	658	0.000
	Yes	74	18.78	8	2.31	82	
Autoimmune and/or connective tissue disease	No	372	94.42	336	97.11	708	0.102
	Yes	22	5.58	10	2.89	32	
Cardiac arrhythmia	No	178	45.18	139	40.17	317	0.181
	Yes	216	54.82	207	59.83	423	
Cerebrovascular disease	No	299	75.89	253	73.12	552	0.398
	Yes	95	24.11	93	26.88	188	
Chronic renal insufficiency	No	230	58.38	190	54.91	420	0.372
	Yes	164	41.62	156	45.09	320	
CNS neurological disease	No	383	97.21	325	93.93	708	0.031
	Yes	11	2.79	21	6.07	32	
COPD	No	304	77.16	163	47.11	467	0.000
	Yes	90	22.84	183	52.89	273	
Coronary ischemic disease	No	336	85.28	284	82.08	620	0.272
	Yes	58	14.72	62	17.92	120	
Degenerative arthropathy	No	166	42.13	189	54.62	355	0.001
	Yes	228	57.87	157	45.38	385	
Dementia	No	291	73.86	270	78.03	561	0.197
	Yes	103	26.14	76	21.97	179	
Diabetes mellitus with organ damage	No	324	82.23	283	81.79	607	0.924
	Yes	70	17.77	63	18.21	133	
Diabetes mellitus without organ damage	No	293	74.37	246	71.10	539	0.322
	Yes	101	25.63	100	28.90	201	
Drug related conditions	No	350	88.83	323	93.35	673	0.039
	Yes	44	11.17	23	6.65	67	
Dyslipidemia	No	205	52.03	175	50.58	380	0.713
	Yes	189	47.97	171	49.42	360	
Fibromyalgia	No	388	98.48	344	99.42	732	0.295
	Yes	6	1.52	2	0.58	8	
Fracture (excluding hip)	No	307	77.92	294	84.97	601	0.014
	Yes	87	22.08	52	15.03	139	
Gallstones	No	355	90.10	306	88.44	661	0.476
	Yes	39	9.90	40	11.56	79	
Gastroesophageal reflux disease	No	340	86.29	306	88.44	646	0.439
	Yes	54	13.71	40	11.56	94	
Gout	No	337	85.53	266	76.88	603	0.003
	Yes	57	14.47	80	23.12	137	
Heart failure	No	139	35.28	158	45.66	297	0.004
	Yes	255	64.72	188	54.34	443	
Hematological diseases	No	380	96.45	324	93.64	704	0.088
	Yes	14	3.55	22	6.36	36	
Hip fracture	No	342	86.80	331	95.66	673	0.000
	Yes	52	13.20	15	4.34	67	
Hypertension	No	64	16.24	73	21.10	137	0.107
	Yes	330	83.76	273	78.90	603	
Inflammatory osteoarticular disease	No	368	93.40	323	93.35	691	1.000
	Yes	26	6.60	23	6.65	49	
Irritable bowel syndrome	No	387	98.22	342	98.84	729	0.555
	Yes	7	1.78	4	1.16	11	
Migraine	No	390	98.98	346	100.00	736	0.127
	Yes	4	1.02	0	0.00	4	
Mild liver disease	No	382	96.95	326	94.22	708	0.073
	Yes	12	3.05	20	5.78	32	
Myocardial infarction	No	347	88.07	282	81.50	629	0.013
	Yes	47	11.93	64	18.50	111	

Neoplasia	No	365	92.64	264	76.30	629	0.000
	Yes	29	7.36	82	23.70	111	
Non-ischemic heart disease	No	269	68.27	233	67.34	502	0.813
	Yes	125	31.73	113	32.66	238	
Non-schizophrenic mental disorders	No	383	97.21	345	99.71	728	0.007
	Yes	11	2.79	1	0.29	12	
Obesity	No	267	67.77	280	80.92	547	0.000
	Yes	127	32.23	66	19.08	193	
Osteoporosis	No	312	79.19	325	93.93	637	0.000
	Yes	82	20.81	21	6.07	103	
Other neurological pathologies	No	391	99.24	340	98.27	731	0.317
	Yes	3	0.76	6	1.73	9	
Pancreas disease	No	390	98.98	340	98.27	730	0.528
	Yes	4	1.02	6	1.73	10	
Parkinson's disease	No	379	96.19	327	94.51	706	0.295
	Yes	15	3.81	19	5.49	34	
Peripheral arteriopathy	No	363	92.13	272	78.61	635	0.000
	Yes	31	7.87	74	21.39	105	
Peripheral neuropathy	No	367	93.15	312	90.17	679	0.180
	Yes	27	6.85	34	9.83	61	
Post-traumatic stress disorder	No	393	99.75	344	99.42	737	0.602
	Yes	1	0.25	2	0.58	3	
Schizophrenia	No	393	99.75	344	99.42	737	0.602
	Yes	1	0.25	2	0.58	3	
Severe liver disease	No	385	97.72	336	97.11	721	0.647
	Yes	9	2.28	10	2.89	19	
Sleep apnea	No	360	91.37	314	90.75	674	0.797
	Yes	34	8.63	32	9.25	66	
Thyroid disease	No	301	76.40	304	87.86	605	0.000
	Yes	93	23.60	42	12.14	135	
Tuberculosis	No	389	98.73	342	98.84	731	1.000
	Yes	5	1.27	4	1.16	9	
Ulcerative disease	No	383	97.21	311	89.88	694	0.000
	Yes	11	2.79	35	10.12	46	
Urinary tract stones	No	385	97.72	341	98.55	726	0.433
	Yes	9	2.28	5	1.45	14	
Varicose veins	No	296	75.13	280	80.92	576	0.063
	Yes	98	24.87	66	19.08	164	
Vertigo	No	340	86.29	323	93.35	663	0.002
	Yes	54	13.71	23	6.65	77	

Table S2. Prevalence of chronic conditions stratified by age group 65-84 years. Fisher's exact test. CNS: central nervous system. COPD: chronic obstructive pulmonary disease.

Variable	Level	Female		Male		Total	P-value
		N	%	N	%	N	
Amputation	No	154	98.09	178	95.19	332	0.237
	Yes	3	1.91	9	4.81	12	
Anemia	No	74	47.13	97	51.87	171	0.389
	Yes	83	52.87	90	48.13	173	
Asthma	No	123	78.34	182	97.33	305	0.000
	Yes	34	21.66	5	2.67	39	
Autoimmune and/or connective tissue disease	No	148	94.27	179	95.72	327	0.621
	Yes	9	5.73	8	4.28	17	
Cardiac arrhythmia	No	72	45.86	83	44.39	155	0.828
	Yes	85	54.14	104	55.61	189	
Cerebrovascular disease	No	123	78.34	136	72.73	259	0.259
	Yes	34	21.66	51	27.27	85	
Chronic renal insufficiency	No	92	58.60	105	56.15	197	0.663
	Yes	65	41.40	82	43.85	147	
CNS neurological disease	No	150	95.54	176	94.12	326	0.632
	Yes	7	4.46	11	5.88	18	
COPD	No	126	80.25	93	49.73	219	0.000
	Yes	31	19.75	94	50.27	125	
Coronary ischemic disease	No	132	84.08	156	83.42	288	0.885
	Yes	25	15.92	31	16.58	56	
Degenerative arthropathy	No	59	37.58	104	55.61	163	0.001
	Yes	98	62.42	83	44.39	181	
Dementia	No	122	77.71	151	80.75	273	0.506
	Yes	35	22.29	36	19.25	71	
Diabetes mellitus with organ damage	No	120	76.43	148	79.14	268	0.602
	Yes	37	23.57	39	20.86	76	
Diabetes mellitus without organ damage	No	110	70.06	128	68.45	238	0.815
	Yes	47	29.94	59	31.55	106	
Drug related conditions	No	131	83.44	172	91.98	303	0.019
	Yes	26	16.56	15	8.02	41	
Dyslipidemia	No	75	47.77	79	42.25	154	0.328
	Yes	82	52.23	108	57.75	190	
Fibromyalgia	No	153	97.45	185	98.93	338	0.418
	Yes	4	2.55	2	1.07	6	
Fracture (excluding hip)	No	120	76.43	152	81.28	272	0.289
	Yes	37	23.57	35	18.72	72	
Gallstones	No	141	89.81	165	88.24	306	0.731
	Yes	16	10.19	22	11.76	38	
Gastroesophageal reflux disease	No	135	85.99	168	89.84	303	0.317
	Yes	22	14.01	19	10.16	41	
Gout	No	131	83.44	147	78.61	278	0.274
	Yes	26	16.56	40	21.39	66	
Heart failure	No	54	34.39	90	48.13	144	0.012
	Yes	103	65.61	97	51.87	200	
Hematological diseases	No	151	96.18	173	92.51	324	0.171
	Yes	6	3.82	14	7.49	20	
Hip fracture	No	144	91.72	179	95.72	323	0.174
	Yes	13	8.28	8	4.28	21	
Hypertension	No	25	15.92	38	20.32	63	0.329
	Yes	132	84.08	149	79.68	281	
Inflammatory osteoarticular disease	No	145	92.36	170	90.91	315	0.699
	Yes	12	7.64	17	9.09	29	
Irritable bowel syndrome	No	153	97.45	186	99.47	339	0.182
	Yes	4	2.55	1	0.53	5	
Migraine	No	153	97.45	187	100.00	340	0.042
	Yes	4	2.55	0	0.00	4	
Mild liver disease	No	146	92.99	176	94.12	322	0.826
	Yes	11	7.01	11	5.88	22	
Myocardial infarction	No	136	86.62	150	80.21	286	0.148
	Yes	21	13.38	37	19.79	58	
Neoplasia	No	144	91.72	138	73.80	282	0.000

	Yes	13	8.28	49	26.20	62	
Non-ischemic heart disease	No	107	68.15	118	63.10	225	0.363
	Yes	50	31.85	69	36.90	119	
Non-schizophrenic mental disorders	No	151	96.18	186	99.47	337	0.050
	Yes	6	3.82	1	0.53	7	
Obesity	No	91	57.96	146	78.07	237	0.000
	Yes	66	42.04	41	21.93	107	
Osteoporosis	No	124	78.98	170	90.91	294	0.002
	Yes	33	21.02	17	9.09	50	
Other neurological pathologies	No	156	99.36	184	98.40	340	0.629
	Yes	1	0.64	3	1.60	4	
Pancreas disease	No	157	100.00	183	97.86	340	0.129
	Yes	0	0.00	4	2.14	4	
Parkinson's disease	No	151	96.18	175	93.58	326	0.337
	Yes	6	3.82	12	6.42	18	
Peripheral arteriopathy	No	142	90.45	142	75.94	284	0.001
	Yes	15	9.55	45	24.06	60	
Peripheral neuropathy	No	137	87.26	164	87.70	301	1.000
	Yes	20	12.74	23	12.30	43	
Post-traumatic stress disorder	No	156	99.36	186	99.47	342	1.000
	Yes	1	0.64	1	0.53	2	
Schizophrenia	No	156	99.36	185	98.93	341	1.000
	Yes	1	0.64	2	1.07	3	
Severe liver disease	No	150	95.54	177	94.65	327	0.805
	Yes	7	4.46	10	5.35	17	
Sleep apnea	No	133	84.71	160	85.56	293	0.879
	Yes	24	15.29	27	14.44	51	
Thyroid disease	No	112	71.34	163	87.17	275	0.000
	Yes	45	28.66	24	12.83	69	
Tuberculosis	No	155	98.73	186	99.47	341	0.594
	Yes	2	1.27	1	0.53	3	
Ulcerative disease	No	152	96.82	169	90.37	321	0.018
	Yes	5	3.18	18	9.63	23	
Urinary tract stones	No	153	97.45	182	97.33	335	1.000
	Yes	4	2.55	5	2.67	9	
Varicose veins	No	102	64.97	146	78.07	248	0.008
	Yes	55	35.03	41	21.93	96	
Vertigo	No	132	84.08	174	93.05	306	0.010
	Yes	25	15.92	13	6.95	38	

Table S3. Prevalence of chronic conditions by sex stratified by age group ≥85 years. Fisher's exact test. CNS: central nervous system. COPD: chronic obstructive pulmonary disease.

Variable	Level	Female		Male		Total	P-value
		N	%	N	%	N	
Amputation	No	236	99.58	156	98.11	392	0.307
	Yes	1	0.42	3	1.89	4	
Anemia	No	138	58.23	97	61.01	235	0.603
	Yes	99	41.77	62	38.99	161	
Asthma	No	197	83.12	156	98.11	353	0.000
	Yes	40	16.88	3	1.89	43	
Autoimmune and/or connective tissue disease	No	224	94.51	157	98.74	381	0.033
	Yes	13	5.49	2	1.26	15	
Cardiac arrhythmia	No	106	44.73	56	35.22	162	0.062
	Yes	131	55.27	103	64.78	234	
Cerebrovascular disease	No	176	74.26	117	73.58	293	0.907
	Yes	61	25.74	42	26.42	103	
Chronic renal insufficiency	No	138	58.23	85	53.46	223	0.354
	Yes	99	41.77	74	46.54	173	
CNS neurological disease	No	233	98.31	149	93.71	382	0.023
	Yes	4	1.69	10	6.29	14	
COPD	No	178	75.11	70	44.03	248	0.000
	Yes	59	24.89	89	55.97	148	
Coronary ischemic disease	No	204	86.08	128	80.50	332	0.164
	Yes	33	13.92	31	19.50	64	
Degenerative arthropathy	No	107	45.15	85	53.46	192	0.124
	Yes	130	54.85	74	46.54	204	
Dementia	No	169	71.31	119	74.84	288	0.490
	Yes	68	28.69	40	25.16	108	
Diabetes mellitus with organ damage	No	204	86.08	135	84.91	339	0.771
	Yes	33	13.92	24	15.09	57	
Diabetes mellitus without organ damage	No	183	77.22	118	74.21	301	0.549
	Yes	54	22.78	41	25.79	95	
Drug related conditions	No	219	92.41	151	94.97	370	0.409
	Yes	18	7.59	8	5.03	26	
Dyslipidemia	No	130	54.85	96	60.38	226	0.301
	Yes	107	45.15	63	39.62	170	
Fibromyalgia	No	235	99.16	159	100.00	394	0.518
	Yes	2	0.84	0	0.00	2	
Fracture (excluding hip)	No	187	78.90	142	89.31	329	0.006
	Yes	50	21.10	17	10.69	67	
Gallstones	No	214	90.30	141	88.68	355	0.617
	Yes	23	9.70	18	11.32	41	
Gastroesophageal reflux disease	No	205	86.50	138	86.79	343	1.000
	Yes	32	13.50	21	13.21	53	
Gout	No	206	86.92	119	74.84	325	0.003
	Yes	31	13.08	40	25.16	71	
Heart failure	No	85	35.86	68	42.77	153	0.173
	Yes	152	64.14	91	57.23	243	
Hematological diseases	No	229	96.62	151	94.97	380	0.443
	Yes	8	3.38	8	5.03	16	
Hip fracture	No	198	83.54	152	95.60	350	0.000
	Yes	39	16.46	7	4.40	46	
Hypertension	No	39	16.46	35	22.01	74	0.189
	Yes	198	83.54	124	77.99	322	
Inflammatory osteoarticular disease	No	223	94.09	153	96.23	376	0.483
	Yes	14	5.91	6	3.77	20	
Irritable bowel syndrome	No	234	98.73	156	98.11	390	0.688
	Yes	3	1.27	3	1.89	6	
Mild liver disease	No	236	99.58	150	94.34	386	0.002
	Yes	1	0.42	9	5.66	10	
Myocardial infarction	No	211	89.03	132	83.02	343	0.098
	Yes	26	10.97	27	16.98	53	
Neoplasia	No	221	93.25	126	79.25	347	0.000
	Yes	16	6.75	33	20.75	49	
Non-ischemic heart disease	No	162	68.35	115	72.33	277	0.435

	Yes	75	31.65	44	27.67	119	
Non-schizophrenic mental disorders	No	232	97.89	159	100.00	391	0.086
	Yes	5	2.11	0	0.00	5	
Obesity	No	176	74.26	134	84.28	310	0.018
	Yes	61	25.74	25	15.72	86	
Osteoporosis	No	188	79.32	155	97.48	343	0.000
	Yes	49	20.68	4	2.52	53	
Other neurological pathologies	No	235	99.16	156	98.11	391	0.395
	Yes	2	0.84	3	1.89	5	
Pancreas disease	No	233	98.31	157	98.74	390	1.000
	Yes	4	1.69	2	1.26	6	
Parkinson's disease	No	228	96.20	152	95.60	380	0.798
	Yes	9	3.80	7	4.40	16	
Peripheral arteriopathy	No	221	93.25	130	81.76	351	0.001
	Yes	16	6.75	29	18.24	45	
Peripheral neuropathy	No	230	97.05	148	93.08	378	0.084
	Yes	7	2.95	11	6.92	18	
Post-traumatic stress disorder	No	237	100.00	158	99.37	395	0.402
	Yes	0	0.00	1	0.63	1	
Severe liver disease	No	235	99.16	159	100.00	394	0.518
	Yes	2	0.84	0	0.00	2	
Sleep apnea	No	227	95.78	154	96.86	381	0.789
	Yes	10	4.22	5	3.14	15	
Thyroid disease	No	189	79.75	141	88.68	330	0.020
	Yes	48	20.25	18	11.32	66	
Tuberculosis	No	234	98.73	156	98.11	390	0.688
	Yes	3	1.27	3	1.89	6	
Ulcerative disease	No	231	97.47	142	89.31	373	0.001
	Yes	6	2.53	17	10.69	23	
Urinary tract stones	No	232	97.89	159	100.00	391	0.086
	Yes	5	2.11	0	0.00	5	
Varicose veins	No	194	81.86	134	84.28	328	0.588
	Yes	43	18.14	25	15.72	68	
Vertigo	No	208	87.76	149	93.71	357	0.059
	Yes	29	12.24	10	6.29	39	

Table S4. Prevalence of geriatric syndromes by sex for the total cohort. Fisher's exact test.

Variable	Level	Female		Male		Total	P-value
		N	%	N	%	N	
Acute confusional syndrome	No	276	70.05	229	66.18	505	0.269
	Yes	118	29.95	117	33.82	235	
Chronic pain	No	163	41.37	175	50.58	338	0.015
	Yes	231	58.63	171	49.42	402	
Cognitive/Intellectual impairment	No	261	66.24	250	72.25	511	0.080
	Yes	133	33.76	96	27.75	229	
Constipation	No	193	48.98	207	59.83	400	0.004
	Yes	201	51.02	139	40.17	340	
Depression & Anxiety	No	201	51.02	271	78.32	472	0.000
	Yes	193	48.98	75	21.68	268	
Dysphagia	No	316	80.20	265	76.59	581	0.244
	Yes	78	19.80	81	23.41	159	
Polypharmacy	No	84	21.32	65	18.79	149	0.409
	Yes	310	78.68	281	81.21	591	
Immobility	No	280	71.07	268	77.46	548	0.053
	Yes	114	28.93	78	22.54	192	
Instability/falls	No	236	59.90	218	63.01	454	0.406
	Yes	158	40.10	128	36.99	286	
Malnutrition	No	318	80.71	275	79.48	593	0.712
	Yes	76	19.29	71	20.52	147	
Pressure ulcers	No	337	85.53	308	89.02	645	0.186
	Yes	57	14.47	38	10.98	95	
Sensory deficit	No	225	57.11	201	58.09	426	0.823
	Yes	169	42.89	145	41.91	314	
Sleep disorders/Insomnia	No	205	52.03	202	58.38	407	0.089
	Yes	189	47.97	144	41.62	333	
(Urinary/faecal) Incontinence	No	121	30.71	189	54.62	310	0.000
	Yes	273	69.29	157	45.38	430	

Table S5. Potentially inappropriate medication according to STOPP criteria by sex. Fisher's Exact Test.

Variable	Level	Female		Male		Total	P-value
		N	%	N	%	N	
A1: Acid reducer prescribed without an evidence-based clinical indication	No	312	79.19	268	77.46	580	0.592
	Yes	82	20.81	78	22.54	160	
A1: Hypolipidemic drug prescribed without an evidence-based clinical indication	No	379	96.19	332	95.95	711	1.000
	Yes	15	3.81	14	4.05	29	
A1: Analgesic drug prescribed without an evidence-based clinical indication	No	379	96.19	340	98.27	719	0.120
	Yes	15	3.81	6	1.73	21	
A1: Aspirin prescribed without an evidence-based clinical indication	No	382	96.95	342	98.84	724	0.126
	Yes	12	3.05	4	1.16	16	
A1: Antihypertensive drug prescribed without an evidence-based clinical indication	No	388	98.48	342	98.84	730	0.757
	Yes	6	1.52	4	1.16	10	
A1: Other drug prescribed without an evidence-based clinical indication	No	357	90.61	320	92.49	677	0.429
	Yes	37	9.39	26	7.51	63	
A2: Any drug prescribed beyond the recommended duration, where treatment duration is well defined	No	377	95.69	328	94.80	705	0.606
	Yes	17	4.31	18	5.20	35	
A3: Any duplicate drug class prescription	No	378	95.94	333	96.24	711	0.852
	Yes	16	4.06	13	3.76	29	
B1: Digoxin for heart failure with normal systolic ventricular function	No	393	99.75	342	98.84	735	0.191
	Yes	1	0.25	4	1.16	5	
B2: Verapamil or diltiazem with NYHA Class III or IV heart failure	No	390	98.98	345	99.71	735	0.379
	Yes	4	1.02	1	0.29	5	
B3: Beta-blocker in combination with verapamil or diltiazem	No	393	99.75	346	100.00	739	1.000
	Yes	1	0.25	0	0.00	1	
B4: Beta blocker with bradycardia, type II heart block or complete heart block	No	393	99.75	344	99.42	737	0.602
	Yes	1	0.25	2	0.58	3	
B5: Amiodarone as first-line antiarrhythmic therapy in supraventricular tachyarrhythmia	No	388	98.48	339	97.98	727	0.781
	Yes	6	1.52	7	2.02	13	
B6: Loop diuretic as first-line treatment for hypertension	No	388	98.48	339	97.98	727	0.781
	Yes	6	1.52	7	2.02	13	
B7: Loop diuretic for dependent ankle oedema without clinical, biochemical evidence or radiological evidence of heart failure, liver failure, nephrotic syndrome or renal failure	No	390	98.98	342	98.84	732	1.000
	Yes	4	1.02	4	1.16	8	
B8: Thiazide diuretic with current significant hypokalaemia, hyponatraemia, hypercalcaemia or with a history of gout	No	382	96.95	340	98.27	722	0.340
	Yes	12	3.05	6	1.73	18	
B9: Loop diuretic for treatment of hypertension with concurrent urinary incontinence	No	391	99.24	344	99.42	735	1.000
	Yes	3	0.76	2	0.58	5	
B11: ACE inhibitors or Angiotensin Receptor Blockers in patients with hyperkalaemia	No	372	94.42	324	93.64	696	0.756
	Yes	22	5.58	22	6.36	44	
B12: Aldosterone antagonists with concurrent potassium-conserving drugs without monitoring of serum potassium	No	386	97.97	337	97.40	723	0.631
	Yes	8	2.03	9	2.60	17	
C1: Long-term aspirin at doses greater than 160mg per day	No	386	97.97	340	98.27	726	0.795
	Yes	8	2.03	6	1.73	14	
C2: Aspirin with a past history of peptic ulcer disease without concomitant PPI	No	392	99.49	344	99.42	736	1.000
	Yes	2	0.51	2	0.58	4	
C3: Aspirin, clopidogrel, dipyridamole, vitamin K antagonists, direct thrombin inhibitors or factor Xa inhibitors with concurrent significant bleeding risk	No	389	98.73	346	100.00	735	0.064
	Yes	5	1.27	0	0.00	5	
C4: Aspirin plus clopidogrel as secondary stroke prevention, unless the patient has a coronary stent(s) inserted in the previous 12 months or concurrent acute coronary syndrome or has a high grade symptomatic carotid arterial stenosis	No	392	99.49	345	99.71	737	1.000
	Yes	2	0.51	1	0.29	3	
C5: Aspirin in combination with vitamin K antagonist, direct thrombin inhibitor or factor Xa inhibitors in patients with chronic atrial fibrillation	No	390	98.98	342	98.84	732	1.000
	Yes	4	1.02	4	1.16	8	
C6: Antiplatelet agents with vitamin K antagonist, direct thrombin inhibitor or factor Xa inhibitors in patients with stable coronary, cerebrovascular or peripheral arterial disease	No	393	99.75	345	99.71	738	1.000
	Yes	1	0.25	1	0.29	2	
C8: Vitamin K antagonist, direct thrombin inhibitor or factor Xa inhibitors for first deep venous thrombosis without continuing provoking risk factors for > 6 months	No	393	99.75	345	99.71	738	1.000
	Yes	1	0.25	1	0.29	2	
C9: Vitamin K antagonist, direct thrombin inhibitor or factor Xa inhibitors for first pulmonary embolus without continuing provoking risk factors for > 12 months	No	392	99.49	345	99.71	737	1.000
	Yes	2	0.51	1	0.29	3	
C10: NSAID and vitamin K antagonist, direct thrombin inhibitor or factor Xa inhibitors in combination	No	390	98.98	345	99.71	735	0.379
	Yes	4	1.02	1	0.29	5	
	No	389	98.73	344	99.42	733	0.458

D1: TriCyclic Antidepressants (TCAs) with dementia, narrow angle glaucoma, cardiac conduction abnormalities, prostatism, or prior history of urinary retention	Yes	5	1.27	2	0.58	7	
D2: Initiation of TriCyclic Antidepressants (TCAs) as first-line antidepressant treatment	No	392	99.49	345	99.71	737	1.000
	Yes	2	0.51	1	0.29	3	
D3: Neuroleptics with moderate-marked antimuscarinic/anticholinergic effects with a history of prostatism or previous urinary retention	No	394	100.00	344	99.42	738	0.218
	Yes	0	0.00	2	0.58	2	
D4: Selective serotonin re-uptake inhibitors (SSRI's) with current or recent significant hyponatraemia i.e. serum Na+ < 130 mmol/l	No	389	98.73	346	100.00	735	0.064
	Yes	5	1.27	0	0.00	5	
D5: Benzodiazepines for 4 or more weeks	No	251	63.71	254	73.41	505	0.006
	Yes	143	36.29	92	26.59	235	
D7: Anticholinergics/antimuscarinics to treat extra-pyramidal side-effects of neuroleptic medications	No	393	99.75	346	100.00	739	1.000
	Yes	1	0.25	0	0.00	1	
D8: Anticholinergics/antimuscarinics in patients with delirium or dementia	No	391	99.24	341	98.55	732	0.484
	Yes	3	0.76	5	1.45	8	
D9: Neuroleptic antipsychotic in patients with behavioural and psychological symptoms of dementia (BPSD) unless symptoms are severe and other non-pharmacological treatments have failed	No	392	99.49	345	99.71	737	1.000
	Yes	2	0.51	1	0.29	3	
D10: Neuroleptics as hypnotics, unless sleep disorder is due to psychosis or dementia	No	393	99.75	345	99.71	738	1.000
	Yes	1	0.25	1	0.29	2	
D11: Acetylcholinesterase inhibitors with a known history of persistent bradycardia, heart block or recurrent unexplained syncope or concurrent treatment with drugs that reduce heart rate	No	394	100.00	345	99.71	739	0.468
	Yes	0	0.00	1	0.29	1	
D14: First-generation antihistamines	No	392	99.49	345	99.71	737	1.000
	Yes	2	0.51	1	0.29	3	
E1: Digoxin at a long-term dose greater than 125µg/day if eGFR < 30 ml/min/1.73m2	No	393	99.75	344	99.42	737	0.602
	Yes	1	0.25	2	0.58	3	
E4: NSAID's if eGFR < 50 ml/min/1.73m2	No	389	98.73	342	98.84	731	1.000
	Yes	5	1.27	4	1.16	9	
E6: Metformin if eGFR < 30 ml/min/1.73m2	No	388	98.48	343	99.13	731	0.513
	Yes	6	1.52	3	0.87	9	
F2: PPI for uncomplicated peptic ulcer disease or erosive peptic oesophagitis at full therapeutic dosage for > 8 weeks	No	389	98.73	344	99.42	733	0.458
	Yes	5	1.27	2	0.58	7	
F3: Drugs likely to cause constipation in patients with chronic constipation where non-constipating alternatives are available	No	394	100.00	345	99.71	739	0.468
	Yes	0	0.00	1	0.29	1	
F4: Oral elemental iron doses greater than 200 mg daily	No	392	99.49	346	100.00	738	0.501
	Yes	2	0.51	0	0.00	2	
G2: Systemic corticosteroids instead of inhaled corticosteroids for maintenance therapy in moderate-severe COPD	No	394	100.00	344	99.42	738	0.218
	Yes	0	0.00	2	0.58	2	
G3: Anti-muscarinic bronchodilators (e.g. ipratropium, tiotropium) with a history of narrow angle glaucoma or bladder outflow obstruction	No	393	99.75	345	99.71	738	1.000
	Yes	1	0.25	1	0.29	2	
G4: Non-selective beta-blocker (whether oral or topical for glaucoma) with a history of asthma requiring treatment	No	391	99.24	345	99.71	736	0.627
	Yes	3	0.76	1	0.29	4	
G5: Benzodiazepines with acute or chronic respiratory failure i.e. pO2 < 8.0 kPa ± pCO2 > 6.5 kPa	No	368	93.40	321	92.77	689	0.772
	Yes	26	6.60	25	7.23	51	
H2: NSAID with severe hypertension or severe heart failure	No	390	98.98	345	99.71	735	0.379
	Yes	4	1.02	1	0.29	5	
H3: Long-term use of NSAID (>3 months) for symptom relief of osteoarthritis pain where paracetamol has not been tried	No	393	99.75	346	100.00	739	1.000
	Yes	1	0.25	0	0.00	1	
H5: Corticosteroids (other than periodic intra-articular injections for mono-articular pain) for osteoarthritis	No	393	99.75	346	100.00	739	1.000
	Yes	1	0.25	0	0.00	1	
H7: COX-2 selective NSAIDs with concurrent cardiovascular disease	No	393	99.75	346	100.00	739	1.000
	Yes	1	0.25	0	0.00	1	
I1: Antimuscarinic drugs with dementia, or chronic cognitive impairment or narrow-angle glaucoma, or chronic prostatism	No	389	98.73	339	97.98	728	0.562
	Yes	5	1.27	7	2.02	12	
I2: Selective alpha-1 selective alpha blockers in those with symptomatic orthostatic hypotension or micturition syncope	No	394	100.00	345	99.71	739	0.468
	Yes	0	0.00	1	0.29	1	
J1: Sulphonylureas with a long duration of action (e.g. glibenclamide, chlorpropamide, glimepiride) with type 2 diabetes mellitus	No	392	99.49	344	99.42	736	1.000
	Yes	2	0.51	2	0.58	4	
J3: Beta-blockers in diabetes mellitus with frequent hypoglycaemic episodes	No	391	99.24	346	100.00	737	0.252
	Yes	3	0.76	0	0.00	3	
K1: Benzodiazepines	No	319	80.96	297	85.84	616	0.093
	Yes	75	19.04	49	14.16	124	
K2: Neuroleptic drugs	No	374	94.92	336	97.11	710	0.140
	Yes	20	5.08	10	2.89	30	
	No	392	99.49	343	99.13	735	0.669

K3: Vasodilator drugs with persistent postural hypotension i.e. recurrent drop in systolic blood pressure ≥ 20mmHg	Yes	2	0.51	3	0.87	5	
K4: Hypnotic Z-drugs e.g. zopiclone, zolpidem, zaleplon	No	388	98.48	341	98.55	729	1.000
	Yes	6	1.52	5	1.45	11	
L1: Use of oral or transdermal strong opioids as first line therapy for mild pain	No	381	96.70	343	99.13	724	0.024
	Yes	13	3.30	3	0.87	16	
L2: Use of regular (as distinct from PRN) opioids without concomitant laxative	No	371	94.16	331	95.66	702	0.406
	Yes	23	5.84	15	4.34	38	
L3: Long-acting opioids without short-acting opioids for break-through pain	No	391	99.24	346	100.00	737	0.252
	Yes	3	0.76	0	0.00	3	
N1: Concomitant use of two or more drugs with antimuscarinic/anticholinergic properties	No	393	99.75	346	100.00	739	1.000
	Yes	1	0.25	0	0.00	1	

Table S6. Potential prescribing omissions according to START criteria by sex. Fisher's Exact Test.

Variable	Level	Female N	%	Male N	%	Total N	P-value
A1: Vitamin K antagonists or direct thrombin / factor Xa inhibitors in the presence of chronic atrial fibrillation	No	383	97.21	337	97.40	720	1.000
	Yes	11	2.79	9	2.60	20	
A2: Aspirin in the presence of chronic atrial fibrillation, where Vitamin K antagonists or direct thrombin / factor Xa inhibitors are contraindicated	No	393	99.75	345	99.71	738	1.000
	Yes	1	0.25	1	0.29	2	
A3: Antiplatelet therapy with a documented history of coronary, cerebral or peripheral vascular disease	No	391	99.24	340	98.27	731	0.317
	Yes	3	0.76	6	1.73	9	
A4: Antihypertensive therapy where systolic blood pressure consistently > 160 mmHg and/or diastolic blood pressure consistently >90 mmHg; if systolic blood pressure > 140 mmHg and /or diastolic blood pressure > 90 mmHg, if diabetic	No	391	99.24	340	98.27	731	0.317
	Yes	3	0.76	6	1.73	9	
A5: Statin therapy with a documented history of coronary, cerebral or peripheral vascular disease, unless the patient's status is end-of-life or age is > 85 years	No	393	99.75	344	99.42	737	0.602
	Yes	1	0.25	2	0.58	3	
A6: ACE inhibitor with systolic heart failure and/or documented coronary artery disease	No	372	94.42	330	95.38	702	0.618
	Yes	22	5.58	16	4.62	38	
A7: Beta-blocker with ischaemic heart disease	No	387	98.22	339	97.98	726	1.000
	Yes	7	1.78	7	2.02	14	
A8: Appropriate beta-blocker (bisoprolol, nebivolol, metoprolol or carvedilol) with stable systolic heart failure	No	369	93.65	332	95.95	701	0.188
	Yes	25	6.35	14	4.05	39	
B1: Regular inhaled b2 agonist or antimuscarinic bronchodilator (e.g. ipratropium, tiotropium) for mild to moderate asthma or COPD	No	394	100.00	344	99.42	738	0.218
	Yes	0	0.00	2	0.58	2	
B2: Regular inhaled corticosteroid for moderate-severe asthma or COPD, where FEV1 <50% of predicted value and repeated exacerbations requiring treatment with oral corticosteroids	No	392	99.49	345	99.71	737	1.000
	Yes	2	0.51	1	0.29	3	
C2: Non-TCA antidepressant drug in the presence of persistent major depressive symptoms	No	387	98.22	339	97.98	726	1.000
	Yes	7	1.78	7	2.02	14	
C3: Acetylcholinesterase inhibitor (e.g. donepezil, rivastigmine, galantamine) for mild-moderate Alzheimer's dementia or Lewy Body dementia (rivastigmine)	No	391	99.24	340	98.27	731	0.317
	Yes	3	0.76	6	1.73	9	
C5: Selective serotonin reuptake inhibitor (or SNRI or pregabalin if SSRI contraindicated) for persistent severe anxiety that interferes with independent functioning	No	392	99.49	346	100.00	738	0.501
	Yes	2	0.51	0	0.00	2	
D1: Proton Pump Inhibitor with severe gastro-oesophageal reflux disease or peptic stricture requiring dilatation	No	392	99.49	342	98.84	734	0.426
	Yes	2	0.51	4	1.16	6	
D2: Fibre supplements (e.g. bran, ispaghula, methylcellulose, sterculia) for diverticulosis with a history of constipation	No	393	99.75	345	99.71	738	1.000
	Yes	1	0.25	1	0.29	2	
E1: Disease-modifying anti-rheumatic drug (DMARD) with active, disabling rheumatoid disease	No	394	100.00	345	99.71	739	0.468
	Yes	0	0.00	1	0.29	1	
E2: Bisphosphonates and vitamin D and calcium in patients taking long-term systemic corticosteroid therapy	No	387	98.22	343	99.13	730	0.351
	Yes	7	1.78	3	0.87	10	
E3: Vitamin D and calcium supplement in patients with known osteoporosis and/or previous fragility fracture(s)	No	379	96.19	340	98.27	719	0.120
	Yes	15	3.81	6	1.73	21	
E4: Bone anti-resorptive or anabolic therapy (e.g. bisphosphonate, strontium ranelate, teriparatide, denosumab) in patients with documented osteoporosis, where no pharmacological or clinical status contraindication exists (Bone Mineral Density T-scores -> 2.5 in multiple sites) and/or previous history of fragility fracture(s).	No	390	98.98	343	99.13	733	1.000
	Yes	4	1.02	3	0.87	7	
E5: Vitamin D supplement in older people who are housebound or experiencing falls or with osteopenia	No	357	90.61	307	88.73	664	0.467
	Yes	37	9.39	39	11.27	76	
E6: Xanthine-oxidase inhibitors (e.g. allopurinol, febuxostat) with a history of recurrent episodes of gout	No	393	99.75	344	99.42	737	0.602
	Yes	1	0.25	2	0.58	3	
E7: Folic acid supplement in patients taking methotexate	No	394	100.00	345	99.71	739	0.468
	Yes	0	0.00	1	0.29	1	
F1: ACE inhibitor or Angiotensin Receptor Blocker (if intolerant of ACE inhibitor) in diabetes with evidence of renal disease i.e. dipstick proteinuria or microalbuminuria (>30mg/24 hours) with or without serum biochemical renal impairment.	No	393	99.75	343	99.13	736	0.345
	Yes	1	0.25	3	0.87	4	
G1: Alpha-1 receptor blocker with symptomatic prostatism, where prostatectomy is not considered necessary	No	394	100.00	344	99.42	738	0.218
	Yes	0	0.00	2	0.58	2	
G2: 5-alpha reductase inhibitor with symptomatic prostatism, where prostatectomy is not considered necessary	No	394	100.00	342	98.84	736	0.047
	Yes	0	0.00	4	1.16	4	
H1: High-potency opioids in moderate-severe pain, where paracetamol, NSAIDs or low-potency opioids are not appropriate to the pain severity or have been ineffective	No	393	99.75	345	99.71	738	1.000
	Yes	1	0.25	1	0.29	2	
H2: Laxatives in patients receiving opioids regularly	No	358	90.86	332	95.95	690	0.008
	Yes	36	9.14	14	4.05	50	
